# E‐SSSentielle‐Fragen und 3‐D‐Eselsbrücke zur Diagnose dermatologischer Erkrankungen: E‐SSSential questions and 3‐D mnemonic to diagnose dermatologic diseases

**DOI:** 10.1111/ddg.70258x

**Published:** 2026-11-10

**Authors:** Falk Ochsendorf

**Affiliations:** ^1^ Goethe Universität Frankfurt Universitätsmedizin Klinik für Dermatologie Venerologie und Allergologie Frankfurt am Main

Sehr geehrte Herausgeber,

Ich habe Salavas Korrespondenz mit großem Interesse gelesen.^1^ Es war erfreulich festzustellen, dass der Ansatz, den ich seit mehr als drei Jahrzehnten praktiziere und sowohl Studierenden als auch Ärzten in der Weiterbildung vermittle,^2^ sich auch für ihn als hilfreich erwiesen hat. Die vorgeschlagene Eselsbrücke „3 C” entspricht meinem eigenen Ansatz, den ich auf Deutsch als „3 S”‐Fragen bezeichne („Seit wann? Symptome? Salben (als Synonym für vorherige Therapie = Heilung)”. Jeder kann selbst entscheiden, welche Eselsbrücke (3 C oder 3 S) er bevorzugt. Wie in der Korrespondenz dargelegt, hat dieser Ansatz mehrere Vorteile, wie beispielsweise eine klare Struktur, eine Gedächtnisstütze für kritische Aspekte der Anamnese und eine leicht zu erlernende Grundlage für Studierende.^1^

Ich möchte jedoch die klinische Relevanz dieser Fragen (Tabelle 1) hervorheben und auf die allgemeineren Aspekte der Vorteile dieses Ansatzes eingehen.

**TABELLE 1:** 3 C/3 S Eselsbrücken und die klinische Relevanz der jeweiligen Fragen.

3 C	3 S	Kann dabei helfen die Erkrankung einzuordnen
**Course**	**Seit wann?**	Akute / chronisch / rezidivierende Erkrankung, Genodermatose und andere
**Complaints**	**Symptome?**	Allergisch/infektiös/neoplastisch und andere
**Cure**	**Salben?**	Primäre Hautveränderungen / Erscheinungsbild durch Vortherapie verändert / Hinweis auf Pathophysiologie abhängig vom Ansprechen auf die Behandlung

1. Warum sollten genau diese drei Fragen gestellt werden?

Weil die Antworten auf diese Fragen nicht aus der klinischen Untersuchung abgeleitet werden können – sie sind nicht sichtbar! Kratzspuren können beispielsweise auf Juckreiz hindeuten, aber sie können auch unabhängig von der Hauterkrankung auftreten und spiegeln nicht unbedingt deren Schweregrad wider. Schüttelfrost/Fieber sind klinisch nicht immer sofort erkennbar. Alles andere lässt sich durch eine Untersuchung der Haut feststellen (siehe 3 D unten;^2^)!

2. Warum sollten zunächst nur diese drei Fragen gestellt werden?

Weil dies vor Fehlern schützt! Im Gegensatz zu anderen Fachgebieten, insbesondere der Inneren Medizin, sollte die erste Anamnese in der Dermatologie kurz sein („zuerst schauen, dann reden”).^2^ Dadurch wird verhindert, dass Hypothesen oder Erklärungen des Patienten beziehungsweise vorbehandelnder Ärzte unbeabsichtigt zu diagnostischer Verzerrung führen.^3^ Hat dies stattgefunden kann es schwierig sein, sich davon zu lösen.

3. Was sind die Vorteile des 3S/3C‐Ansatzes?

Das Verfahren ist zeitsparend! Es ermöglicht einen schnellen direkten Kontakt zum Patienten, bevor die Untersuchung beginnt. Wenn beispielsweise die Befunde auf eine berufsbedingte Kontaktdermatitis hindeuten, ist eine ausführliche Anamnese hilfreich, bei klinischen Befunden, die auf Gürtelrose hindeuten, jedoch nicht notwendig. Daher wird die umfassendere, gezielte Anamnese erst nach der Erfassung der Befunde durchgeführt (siehe 3 D unten).

Zusätzlich zum strukturierten anamnestischen Vorgehen (3S/3C‐Eselsbrücke) möchte ich die folgende 3 D‐Eselsbrücke für die Diagnose dermatologischer Erkrankungen vorschlagen, die sich in der Praxis ebenfalls bewährt hat^2^ (Tabelle 2). Nach den 3S/3C‐Fragen sollten die folgenden Schritte durchgeführt werden:

1. **Deskribieren**. Die Beschreibung der Befunde sollte systematisch und strukturiert erfolgen („vom Großen zum Kleinen“: Lokalisation, Verteilung, Form, Hautveränderungen) und eine allgemein akzeptierte Nomenklatur verwendet werden (siehe^4^ für Englisch und^2^ für Deutsch). Begriffe wie „Hautveränderungen“ oder „Hautläsionen“ sind hier leider nicht hilfreich und sollten vermieden werden!
2. **Differenzieren** (das heißt Differentialdiagnosen (DDs) formulieren/Hypothesen aufstellen). Auf der Grundlage der Informationen aus der 3 S/3 C‐Anamnese und den klinischen Befunden werden Differentialdiagnosen/Hypothesen formuliert: Was muss in der Haut passiert sein, damit der beobachtete Befund im beschriebenen Kontext entstanden ist? Hier ist es hilfreich, „breit” zu denken, das heißt so viele DDs/Hypothesen wie möglich zu formulieren.
3. **Diagnostizieren**/bewusstes Nachdenken (diagnostische Maßnahmen zur Bestätigung oder zum Ausschluss der DDs). Um kognitive Verzerrungen zu vermeiden, ist es ratsam, nach Argumenten *gegen* die anfängliche visuelle Diagnose zu suchen. So kann entweder eine definitive Diagnose gestellt werden oder es bleiben nur noch wenige mögliche Differentialdiagnosen übrig^5^. Dieser Prozess, das bewusste Nachdenken (= *deliberate reflection*), verbessert nachweislich das klinische Denken und die Diagnosegenauigkeit im Allgemeinen,^6^, ^7^ und in der Dermatologie.^5^

**TABELLE 2:** 3 D Eselsbrücke für die Diagnose dermatologischer Erkrankungen^*^

Schritt	Funktion und Vorteile
**Deskribieren**	Zwingt zum genauen Betrachten der Haut („vom Großen zum Kleinen: Lokalisation, Verteilung, Form Effloreszenzen“) Regt kognitive Aktivitäten an: was passiert in der Haut?
**Differenzieren**	Formulieren von Differentialdiagnosen/Hypothesen: welche Erkrankungen könnten diesen Verlauf haben, diese Symptome und diesen Befund? Aktiviert Typ I Denken.^*^
**Diagnostizieren/** **bewusstes Nachdenken (deliberate reflection)**	Ausschließen von Hypothesen, die aus dem einen oder anderen Grund nicht zur vermuteten Differentialdiagnose passen (Anamnese, klinische Befunde); ermitteln der diagnostischen Maßnahmen, um die verbliebenen Hypothesen/DD auszuschließen oder zu bestätigen (wie weitere Anamnese und/oder Labor‐, technische/histologische oder sonstige Untersuchungen). Aktiviert Typ II Denken, um Verzerrungen zu minimieren.^*^

* Typ 1 ist schnell, geschieht automatisch, unbewusst und ohne Anstrengung (intuitiv, heuristisch, „Mustererkennung“); Typ 2 ist langsam, kontrolliert, bewusst und ist anstrengend (reflexiv, analytisch). Details finden sich bei.^2^, ^8^

Die verbleibenden Verdachtsdiagnosen werden nun durch geeignete Maßnahmen bestätigt oder ausgeschlossen, zum Beispiel durch Anamnese, technische Untersuchungen oder Biopsien. Wenn die Diagnose nach Erhalt der Ergebnisse der jeweiligen Tests weiterhin unklar bleibt, ist es ratsam, diese drei Schritte zu wiederholen, beginnend mit einer neuen Beurteilung der Befunde. Meiner Erfahrung nach wird der zweite Schritt, die Suche nach Differentialdiagnosen, oft übersprungen. Darauf folgt häufig eine breite, meist nicht zielgerichtete Diagnostik. Dies führt oft zu pathologischen Befunden, die für den konkreten Fall irrelevant sind, aber weiterer Abklärung bedürfen und manchmal ungünstige Folgen für die Patienten haben können. Die konsequente Einhaltung dieser drei Schritte schützt davor.

Zusammenfassend sollte neben der theoretischen Begründung auch die praktische Relevanz des 3 C‐ oder 3 S‐Ansatzes hervorgehoben werden. Für eine erfolgreiche dermatologische Diagnose wird empfohlen, die 3 C/3 S‐Fragen in der täglichen Praxis durch einen 3 D‐Ansatz zu ergänzen.

## DANKSAGUNG

Open access Veröffentlichung ermöglicht und organisiert durch Projekt DEAL.

## INTERESSENKONFLIKT

Keiner.
